# Body condition and habitat use by Hermann's tortoises in burnt and intact habitats

**DOI:** 10.1093/conphys/cou019

**Published:** 2014-06-12

**Authors:** S. Lecq, J.-M. Ballouard, S. Caron, B. Livoreil, V. Seynaeve, L.-A. Matthieu, X. Bonnet

**Affiliations:** 1 Centre d'Etudes Biologiques de Chizé, CEBC-CNRS UPR 1934, 79360 Villiers en Bois, France; 2 Station d'Observation et de Protection des Tortues et de leurs Milieux, Centre de Recherche et de Conservation des Chéloniens (CRCC), 83590 Gonfaron, France; 3 FRB, 195 rue Saint Jacques, 75005 Paris, France

**Keywords:** Fire mortality, habitat, Mediterranean region, reptiles, *Testudo hermanni*

## Abstract

In Mediterranean regions, fires threaten terrestrial tortoises. Nevertheless, varying proportions of adults survive fire; these surviving individuals can play a central role for population recovery. The regions devastated by fire often include important habitat of Hermann's tortoises (*Testudo hermanni hermanni*), so assessing the ability of survivors to persist is essential for conserving the species. Body-condition indices provide an integrative estimate of how well individuals cope with environmental variations and impacts, including fires. Between 2002 and 2009, we monitored Hermann's tortoises in intact and burnt habitats in southeastern France. In summer 2003, a strong fire ravaged half of the surveyed zone, providing an opportunity to compare body condition of tortoises between intact and burnt areas over time. Six years later, the impact of fire on vegetation was still marked; large trees were abundant in the intact area, whereas open shrub vegetation prevailed in the burnt area. In both areas, the mean body condition of tortoises fluctuated over time; however, there were no differences between the two areas. A radio-tracking experiment demonstrated that individuals from each area were residents, and not vagrants commuting between areas. We also assessed changes in body condition and microhabitat use in radio-tracked individuals. We found no significant differences between the tortoises living in the burnt and intact areas, despite subtle differences in habitat use. In conclusion: (i) surviving tortoises in an area ravaged by fire can maintain their body condition like individuals living in an intact area, and thus, individuals from burnt areas should not be translocated to supposedly better areas; and (ii) depopulated burnt areas are likely to be appropriate for population-augmentation programmes.

## Abstract (precis)

Herman tortoises can maintain their mean body condition in similar range of inter-annual variations in severely burnt versus intact habitats. Therefore, burnt areas provide sufficient food, shelter, and thermally buffered habitats to meet the eco-physiological requirements of the tortoises. Burnt habitats are thus suitable for population reinforcement programs.

## Introduction

In combination with other threats (e.g. illegal collection), the drastic loss and fragmentation of habitat threaten populations of the Hermann's tortoise (*Testudo hermanni hermanni*; Cheylan *et al.*, 2009). As the most favourable areas for *T. hermanni hermanni* are under strong anthropogenic pressures from rapid urbanization, including construction of highways and railways (Debussche *et al.*, 1999; Livoreil, 2009), the less-urbanized habitat areas are necessary for species conservation. In 2003, 380 fires devastated 18 813 hectares of hilly and mountainous areas of south-eastern France, destroying 20% of the native forests and almost 10% of the *T. hermanni* habitat (Prométhée, 2010). Therefore, it is critical that we assess these areas for their suitability in providing key resources, notably food and shelter, to Hermann's tortoises.

In Mediterranean regions, immediate mortality due to fire affects tortoise populations with variable intensity depending upon topography, forest density and season (Hailey, 2000; Sanz-Aguilar *et al.*, 2011). Previous studies showed that the mortality rate ranged between 30% in open landscapes of Spain (Felix *et al.*, 1989) to 88% in wooded habitats of France (Cheylan, 2001). The survivors are essential for population recovery, and fire intensity and frequency are major determinants of whether populations can persist (Sanz-Aguilar *et al.*, 2011). In the present study, we focused on a complementary issue, identifying the suitability of burnt habitat for tortoises by using body condition as an index of the physiological and health status of individuals surviving fires (Pinter-Wollman *et al.*, 2009).

Body condition, i.e. body mass scaled by body size, provides information regarding nutritional and physiological status [body reserves + ovarian follicles + stomach (gut) content + stored water] in vertebrates (Speakman, 2001), including tortoises (Nagy and Medica, 1986; Henen, 1991, 1997; Lagarde *et al.*, 2002). In reptiles, when nutrient resources are abundant, females build up important body reserves and are more likely to undertake vitellogenesis (Naulleau and Bonnet, 1996; Henen, 2002), and they exhibit higher survival rates (Bonnet *et al.*, 1999). Tortoise body condition may decline after fires, because most fires are ignited in summer (Prométhée, 2010), during part of the tortoise active season, and burnt habitats may limit food availability. Also, the massive destruction of vegetation probably reduces the availability of shelter, increases predation risk, perturbs thermoregulation and may generate deleterious chronic stress, causing excessive energy expenditure, thereby reducing body condition (Esque *et al.*, 2003; Bonnet *et al.*, 2013). Overall, burnt habitats may directly and indirectly expose tortoises to a strong degradation of their body condition, with negative consequences on reproductive rate and, ultimately, on populations.

Conversely, tortoises may adjust well to burnt habitats, finding enough food and suitable refuges. Tortoises are low-energy specialists, displaying marked tolerance to fasting and bet-hedging reproductive strategies in unpredictable environments (Henen, 1997, 2002; Lagarde *et al.*, 2003). Thus, we assessed the possible impact of the modification of habitats caused by fire on tortoises. For this, we surveyed tortoise populations in burnt and intact areas 1 year before a strong fire (2002), immediately before (2003) and for the ensuing 5 years (2004–2009). We compared the mean body condition of the tortoises sampled in the two areas. Although straightforward, this comparison was limited by an important potential methodological bias. Individuals captured in one area (intact or burnt) may originate from neighbouring intact or burnt areas. Tortoises can travel over long distances, and the limit created by fire does not necessarily correspond to the natural boundaries of their home ranges. Including vagrant tortoises that commute between areas in the analyses may mix individuals using various habitats and thus generate spurious results. It is therefore important to consider this potential problem. Long-term mark–recapture and radio-tracking surveys are appropriate tools for such assessment. Alternatively, homing behaviour can provide key information; displaced tortoises should rapidly attempt to return home (Chelazzi and Francisci, 1979). In our study, we used this philopatric behaviour to enable better interpretation of the results regarding body condition. In addition, we radio-tracked tortoises in order to provide a more detailed assessment of the impact of fire on habitat use and changes in body condition during the main active period in spring.

## Materials and methods

### Study species and sites

Hermann's tortoise exhibits the typical life-history traits of terrestrial chelonians, including delayed maturity, low fecundity and, thus, low renewal rate (Cheylan, 1981, 2001; Bertolero *et al.*, 2011). Hermann's tortoises do not burrow, rather they tend to shelter beneath thick (and inflammable) shrubs (Cheylan, 1981). The study site (80 ha) is situated in the Massif des Maures in France (Var 83-district, 43°25′N, 6°28′E), a hilly region characterized by dense scrubland ‘maquis’ with cork oak (*Quercus suber*) and pine (*Pinus pinea*) forests. This sclerophyllic vegetation and the extremely powerful wind regimens (e.g. Mistral) make this habitat highly prone to fire (Pausas *et al.*, 2008; Syphard *et al.*, 2009).

The geological substrates are homogeneous across the 80 ha area, which is characterized by a natural, thick maquis and forests, where tortoises have been monitored since 2002. More than 30 years ago (1969), a fire moderately impacted the area (<15% of the surface was burnt), and in 2002, the 80 ha study site was considered as a typical mature maquis (B. Livoreil, unpublished observations). Indeed, in southeast France, the regrowth of mature maquis following a strong fire requires ∼20–30 years (Prométhée, 2010). In July 2003, a very strong fire destroyed half of the area (>90% of the vegetation was burnt, and three fire fighters died; Prométhée, 2010). However, half of the study site was spared, with a road delimiting the two halves into two adjacent areas of ∼40 ha each and named intact vs. burnt areas. In each area, we monitored tortoise body condition over 8 years (2002–2009), assessed vegetation cover and performed a homing-behaviour experiment in 2009. Although the two areas are separated by a road, they are connected to surrounding areas, and tortoises can move from one area to another. Hermann's tortoises will cross roads, as indicated by reports of road mortality (Tok *et al.*, 2011; Gagno *et al.*, 2013).

### Vegetation and ambient temperatures

In 2009, after 5 years of vegetation regrowth, we assessed the vegetation cover of each area using satellite images (Google Earth, 2009). We randomly selected three 2500 m^2^ squares in each area, then we divided each square into smaller 25 m^2^ squares (*n* = 100). In each small square, we estimated by eye the surface covered by trees (i.e. canopy), shrubs and lower layers. The crowns of large trees (crown diameter >8 m) were clearly visible; smaller trees (crown diameter <8 m) were also easily visible. The surface covered by the trees provided an index of the ground surface covered by the canopy. We also estimated the proportion of the surface represented by bare/herbaceous substrate and by shrubs.

During thermoregulation, tortoises (*Testudo* spp.) navigate between open habitats and thick bushes or trees (Lagarde *et al.*, 2012; Attum *et al.*, 2013). Therefore, to assess the thermal suitability of each area, we placed four physical models per area (i.e. operant temperature models constructed from empty tortoise shells filled with a hydro-gel and placed on the ground; see Lagarde *et al.*, 2012; Moulherat *et al.*, 2014 for details) fitted with a temperature logger (Maxim's iButton^®^ device, Thermochron^®^; Maxim Integrated Products, Dallas, TX, USA; accuracy ±0.5°C, glued on the back and recording one measurement every 30 min) in the following two microhabitats: (i) open herbaceous layer exposed to sunlight (two models in each area); and (ii) supposedly thermally buffered thick shrubs or large trees (two models in each area; Lagarde *et al.*, 2012). These physical models were deployed between 10 April and 20 May 2009 and were used to examine the range of temperatures available to tortoises in each area.

### Body condition

We assessed the mean body condition of free-ranging tortoises before and after the fire, in both the intact and the burnt areas. Each individual encountered was captured, sexed using plastron and tail shape, permanently marked (using small metallic tags; B. Livoreil, unpublished observations) and released immediately at the place of capture (Livoreil 2009). For most individuals, the body mass (±1 g, using electronic scales, KL-9505 Kologn) and body size [straight carapace length, SCL ±1 mm, using callipers, Horex 200] were measured. Several individuals were not sexed, notably juveniles, and/or were not fully measured (e.g. SCL lacking) due to human error.

Field population surveys were performed annually. Searches were conducted during the tortoises' main active season of the year (mid-April to mid-June) following a standard protocol (Livoreil 2007, 2009). In each intact or burnt area, several (between one and four) randomly localized quadrats of 50 m × 50 m were surveyed by one person. Surveys were performed by experienced researchers (e.g. B.L.) and/or by volunteers. In each quadrat of 50 m × 50 m, one person randomly searched for tortoises by sight for 30 min. Tortoises were processed (e.g. measured) in the field immediately after capture. The time required to measure tortoises in the field was not counted in order to maintain a net searching time of 30 min per quadrat-session. Volunteers were trained during 1 week prior to their participation in the surveys [e.g. tortoise handling, sex identification, Global Positioning System (GPS) positioning]. Participants were distributed alternately across the quadrats and areas, so that possible observer biases were equally distributed. At least 2 weeks elapsed between consecutive surveys in a given area.

### Homing experiment and habitat use

Between 10 April and 20 May 2009, 24 adult males were measured and fitted with telemetric and temperature devices. Twelve tortoises were monitored in each area (intact and burnt). In each group of 12 tortoises, we randomly assigned six individuals to the control group (released at the place of capture) and displaced six others to trigger homing behaviour. Displacements were performed within each area (intact or burnt), but not between areas in order to minimize the likelihood of tortoises being killed if they crossed the road to home. More specifically, in each study area (intact or burnt) we selected three disparate subareas and released two displaced tortoises in each of them (6.3 ± 0.3 ha on average). These subareas were ∼500–600 m straight line distance apart; therefore, this distance represented the distance used to test homing behaviour for the 12 tortoises released away from their capture sites.

Each individual was fitted with an AVM-K16 transmitter and a temperature data logger glued to the carapace to infer body temperature. This equipment represented <10% of the body mass (Lagarde *et al.*, 2008). The tortoises were located three times per day [morning (09.00–12.00 h), around mid-day (12.00–14.00 h) and afternoon (14.00–17.00 h)], randomly changing individual order every day. We used a Garmin GPS to record each location at a resolution of 5 m. The homing experiment was performed between the 10 April and 20 May 2009. The devices were removed at the end of the experiment; the tortoises were weighed again and released at the place of initial capture. We used only adult males so as not to perturb female reproduction.

In order to characterize the microhabitats used by the radio-tracked tortoises, we viewed a circle 1 m in diameter around each fix, in which we visually estimated the ground surface respectively covered by litter (i.e. leaves), bare soil and herbaceous layer (grass). Then, we evaluated the surface covered by different vegetation types, namely shrubs, small trees (<4 m tall) and large trees (>4 m tall). The same procedure (1 m circle) was applied 25 m away from the tortoise fix, in a random direction (using a table of random numbers), to describe a reference microhabitat that we called ‘randomly sampled habitat’.

### Analysis

Body condition was estimated using residual values from a general linear regression (all individuals pooled), with the natural logarithm (ln) of body mass as the dependent variable and ln SCL as the independent variable (Lagarde *et al.*, 2001; Speakman, 2001; Hailey, 2002; Willemsen and Hailey, 2002). Individual body-condition values were therefore expressed as negative or positive values (without units), with the mean value set to zero by definition. The distribution of residual values did not deviate from normality (Shapiro–Wilk test, *P* > 0.05); therefore, we used parametric analyses of variance (general linear model ANOVA) to assess differences in mean body condition between years, areas and groups of radio-tracked tortoises (i.e. factors). We excluded recaptures to avoid pseudo-replication. Body size, body condition, habitat use and, possibly, energy budget differ between the sexes in *Testudo* (Bonnet *et al.*, 2001; Willemsen and Hailey, 2003; Djordjević *et al.*, 2011); thus, we used sex as an additional factor to detect possible sex-specific differences in tortoises from burnt and intact areas. In two years, 2006 and 2008, there was little survey effort and few tortoises were observed; corresponding sample sizes were low, especially when categorical variables were considered simultaneously (e.g. *n* = 3 per cell), and associated analyses of variance were not robust (Lindman, 1974); therefore, we discarded these years from body-condition analyses (although retaining them did not change the outcomes). We reperformed separate analyses disregarding sex to increase the statistical power of several analyses; this did not change the results.

We did not aim specifically to assess thermoregulation or movement patterns. Instead, to examine changes in mean body condition over time (2002–2009), we focused on the impact of vegetation status (intact vs. burnt) on control vs. displaced radio-tracked tortoises. For instance, we examined whether the degraded vegetation of the burnt area imposed thermal or movement constraints that may influence body condition. For each individual, therefore, we averaged the body-temperature values recorded every day between 10.00 and 16.00 h. This time window was selected to represent a period when individuals could have reached their preferred body temperature, within the range of optimal body temperatures (25–30°C) during activity (Huot-Daubremont and Grenot, 1997). We calculated the mean daily distance travelled during the experiment, using daily starting and ending GPS co-ordinates. During periods of cold weather (when body temperature was below 13°C) and at night, the tortoises remained sheltered; these periods were discarded from temperature and movement analyses. Three temperature data loggers malfunctioned and the data were not used. The 24 tortoises were not fitted with electronic devices simultaneously, and several individuals managed to return home in a few days while others did not. These factors (e.g. malfunctioning device, individual homing speed) generated variations in the sample size (e.g. number of fixes per individual) across analyses.

For the comparisons of the vegetation between intact and burnt areas (2009), we calculated the mean value in each area using the three 2500 m^2^ replicates per area (instead of the 100 25 m^2^ squares per area to avoid an inflation in replicate number); proportions were expressed as values ranging between zero and one (using arcsine transformation did not change the results). Temperatures and canopy cover from selected and randomly sampled microhabitats were compared using Wilcoxon tests. Means were expressed ±1 SD unless stated otherwise (e.g. in figures for presentation clarity). Statistics were performed using R (R Development Core Team, 2012).

## Results

### Impact of fire on vegetation

**Figure 1:**
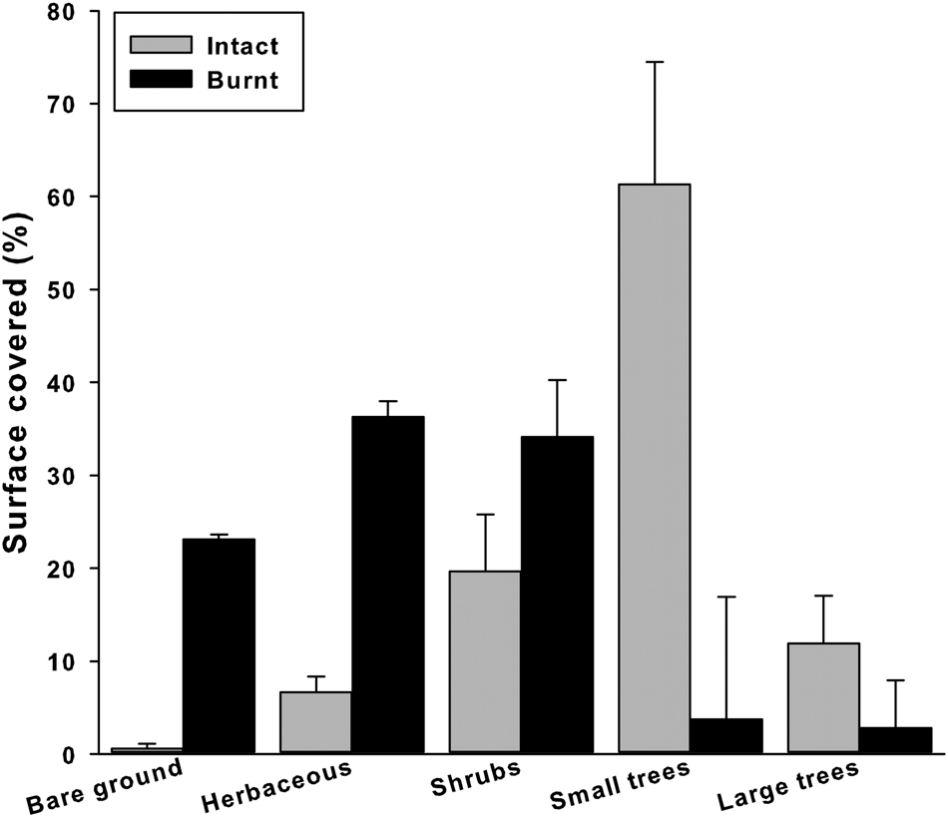
Mean and 1 SE (error bars) percentage cover of the main vegetation types (large trees with a crown wider than 8 m, smaller trees and shrubs) and open ground (herbaceous material, no vegetation) for intact (grey bars) and burnt habitats (black bars) in 2009, 6 years after the 2003 fire. Note that statistics were not performed on percentages, but on values ranging between zero and one (see main text).

In 2009, the respective vegetation cover of the intact and burnt area was significantly different (*F*_4,20_ = 15.27, *P* < 0.001; Fig. 1). Thus, although vegetation appeared to have grown since the 2003 fire, the burnt area was still very different from the unburnt area in 2009. The intact area was relatively closed, with abundant trees and shrubs (Fig. 1), notably oaks, umbrella pines and thick shrub maquis (e.g. heather, *Erica arborea*). In comparison, the vegetation was more open in the burnt area and mostly represented by little shrubs (e.g. *Cistus monspeliensis*) and herbaceous material, with a relatively modest tree cover. Based on the 7500 m^2^ surface assessed in each area, the intact habitat contained 14 large pine trees on average, providing a canopy surface of 3665 m^2^ (272 ± 125 m^2^ per tree). In the burnt area, there were only four pine trees per 7500 m^2^ on average, representing a mean canopy surface of 107 m^2^ (27 ± 8 m^2^ per tree). In both areas, thick shrubs were abundant and provided abundant shelter for tortoises.

**Figure 2:**
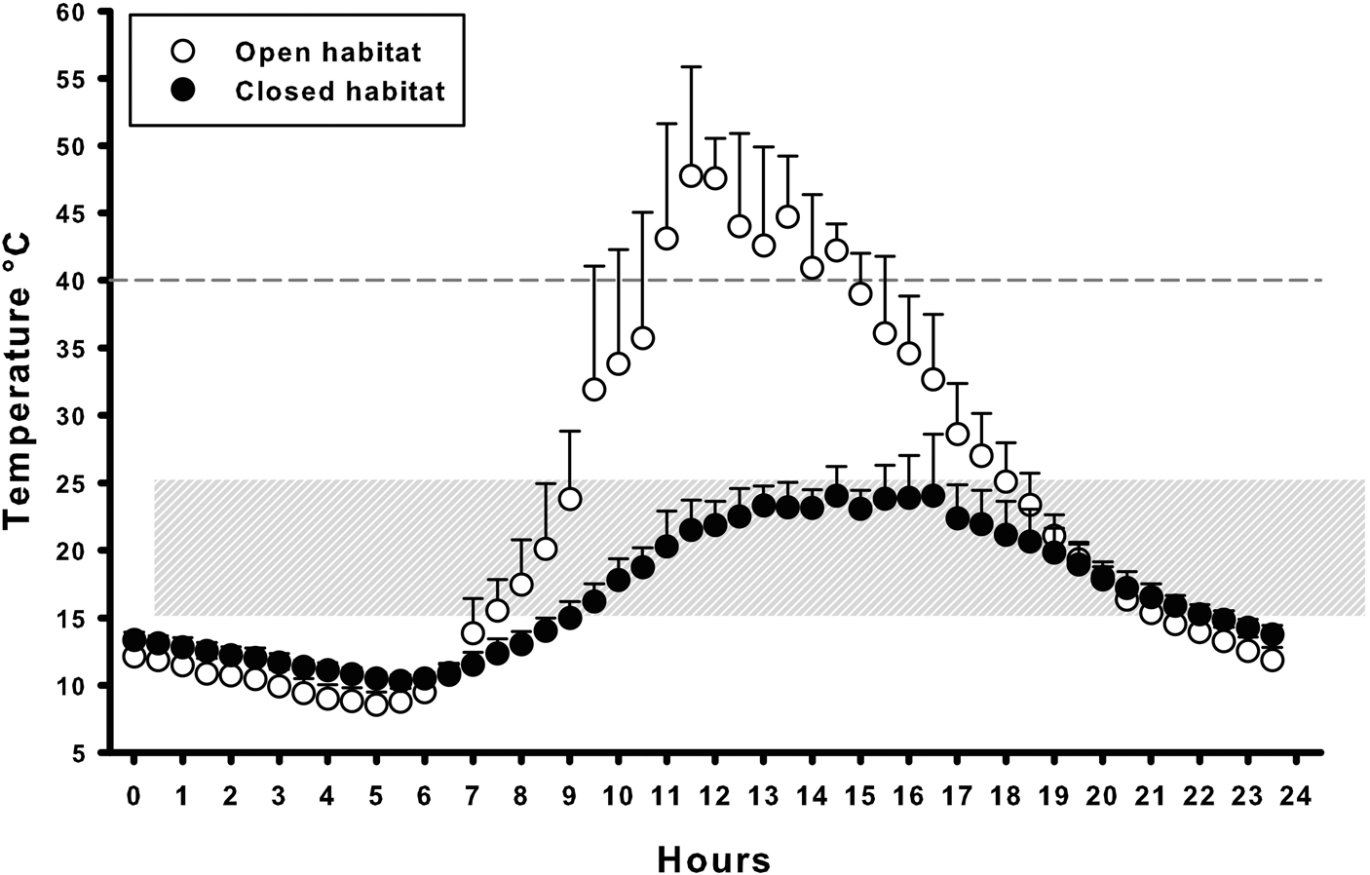
Mean ±1 SD temperatures recorded every 30 min, using physical models, in open microhabitat (herbaceous layer, *n* = 4) and in closed microhabitat (ground beneath large trees, thick shrubs, *n* = 4). Data presented were recorded between April and May 2009, when tortoises are active. The grey shaded area indicates the range of preferred body temperatures during activity in tortoises belonging to the *Testudo* genus; temperatures above 40°C (grey dashed line) can be lethal (Lagarde *et al.*, 2012).

Despite these marked differences, the temperature models indicated that in both areas the available microhabitats (i.e. open zones and thick shrubs or threes) offered a wide range of thermal environments (Fig. 2). In both areas, individuals had ample opportunity to select their preferred body temperatures, and higher or lower temperatures according to their assumed physiological requirements (i.e. ambient temperatures ranging between 20 and 40°C are assumed to meet thermal needs of active tortoises; Lagarde *et al.*, 2012). In other words, tortoises could easily select high body temperatures if needed, could easily escape overheating and could also select intermediate temperatures.

### Body condition: changes over time and impact of fire

**Figure 3:**
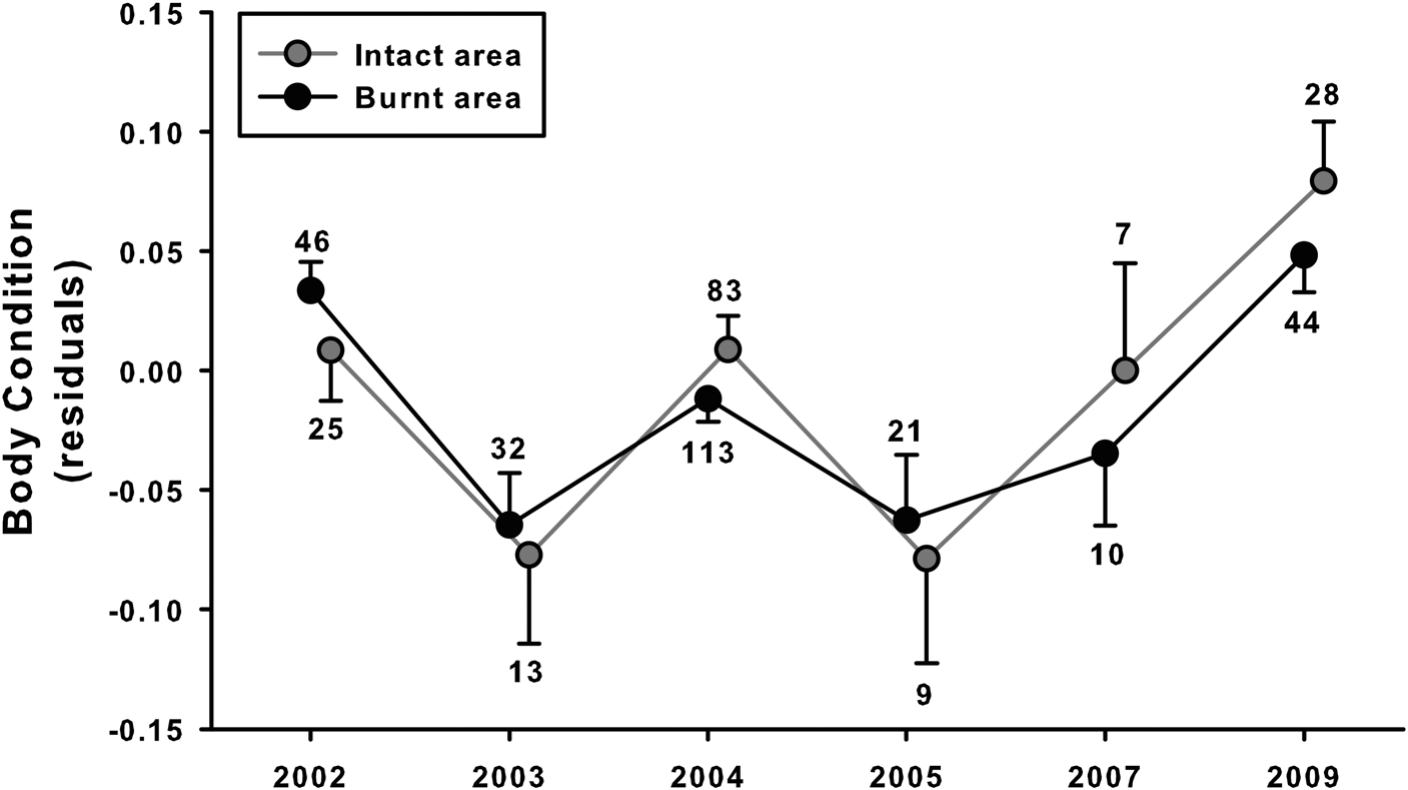
Comparison of mean annual body condition (mass scaled by size; residuals, see main text) of tortoises monitored in two areas, intact and burnt. A major fire occurred in summer 2003 (note that most tortoises, 80%, were assessed before the fire in 2003). Means are expressed ±1 SE; numbers indicate sample size. Due to small sample sizes (*n* < 10 tortoises observed per year), means could not be calculated reliably in 2006 and 2008.

Between 2002 and 2009, 558 adult tortoises were observed during 195 search days. Some information was not properly recorded; therefore, full information (sex, SCL and body mass) was available for only 402 individuals, and body condition for 431 (i.e. 29 individuals were not sexed). The mean body condition of the tortoises varied significantly among years, with no other significant effects (year, *F*_5,378_ = 9.24, *P* < 0.001; sex, *F*_1,378_ = 0.82, *P* = 0.36; area, *F*_1,389_ = 0.13, *P* = 0.72; interaction between the three factors, *F*_5,379_ = 1.20, *P* = 0.31; *P* > 0.50 for other interactions; Fig. 3). Disregarding sex in order to increase statistical power did not change the results; annual variations were not different between the intact and burnt areas (year, *F*_5,419_ = 9.84, *P* < 0.001; area, *F*_1,419_ = 0.13, *P* = 0.72; interaction, *F*_5,419_ = 0.70, *P* = 0.63). *Post hoc* analysis, restricted to year effect in order to avoid an inflation of results (year, *F*_5,425_ = 10.48, *P* < 0.001), suggested that during two years, 2003 and 2005, tortoises exhibited a low mean body-condition index in comparison to the other years (Table 1).

**Table 1:** Summary of the Scheffé *post hoc* tests used to examine differences in mean body condition of tortoises among years (tortoises from intact and burnt areas combined)

	2002	2003	2004	2005	2007
2002					
2003	0.00237*				
2004	0.67298	0.03214*			
2005	0.01573*	1.00000	0.13185		
2007	0.82018	0.81386	0.99608	0.86209	
2009	0.60369	0.00000*	0.00537*	0.00007*	0.21431

Two years, 2003 and 2005, were significantly different from the others.

*Significant probabilities.

### Homing experiment

At the beginning of the homing experiment, the control and displaced tortoises did not differ in body size (area, *F*_1,21_ = 2.89, *P* = 0.10; displacement status, *F*_1,21_ = 1.56, *P* = 0.23; interaction, *F*_1,21_ = 0.11, *P* = 0.74). The control and displaced tortoises also did not differ in body condition (*F*_1,20_ = 0.00, *P* = 0.95; displacement status, *F*_1,20_ = 0.67, *P* = 0.42; interaction, *F*_1,20_ = 1.65, *P* = 0.43).

**Figure 4:**
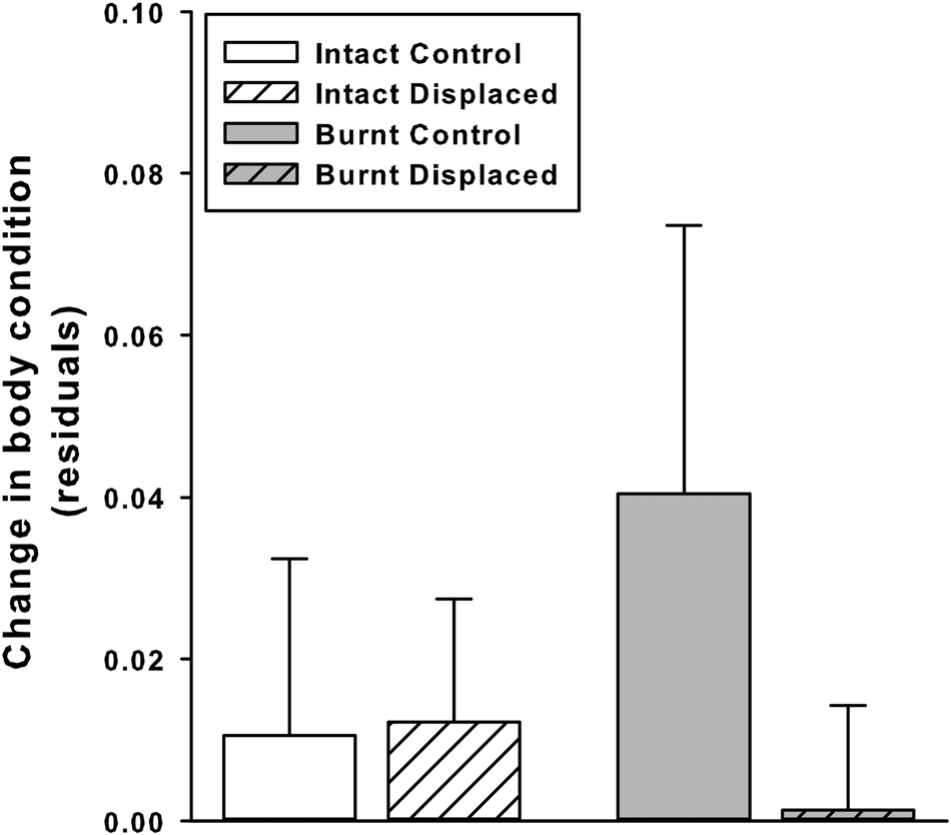
Changes in mean body condition (±1 SE) in control and displaced radio-tracked tortoises in intact and burnt areas, from 10 April to 20 May 2009. Sample size was six for each group.

We observed tortoises foraging during the experiment; however, despite a slight increase in mass we found no significant change between the first and the last body-condition measures (Fig. 4; repeated-measures ANCOVA with successive ln body mass as the dependent variable, ln SCL as a covariate, and area and displacement status as the categorical factors; Wilk-λ = 0.77, *P* = 0.08; specific effect of time, *F*_1,20_ = 0.47, *P* = 0.50).

**Figure 5:**
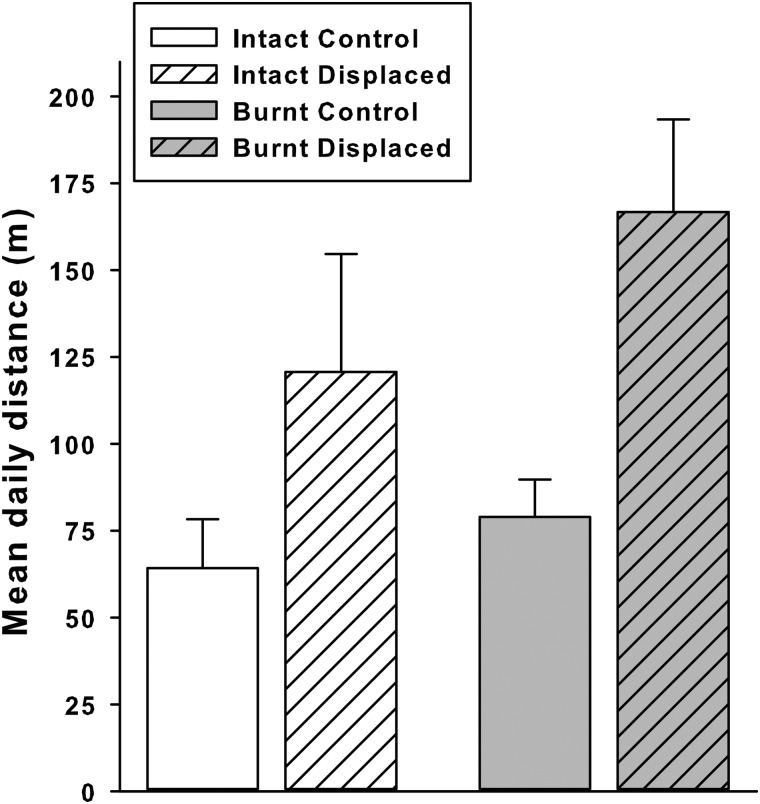
Mean (±1 SE) daily distance travelled by control (*n* = 12; open bars) and displaced radio-tracked tortoises (*n* = 12; hatched bars). Intact and burnt areas are indicated in white and grey, respectively (sample size was six for each group).

None of the 12 control tortoises left their ∼6 ha subarea; in contrast, nine (five in the burnt area and four in the intact area) of the 12 displaced tortoises left the ∼6 ha subarea were they were released and returned home (, *P* < 0.001). The occurrence of homing was not different between the intact and burnt area (, *P* = 1.00). Homing required 3–5 days (mean 4.2 + 1.8 days, *n* = 9) and involved substantial displacements (>500 m straight line; Fig. 5). Thus, displaced tortoises travelled a greater distance between successive fixes compared with control tortoises (*F*_1,20_ = 9.54, *P* < 0.01; Fig. 5). However, this effect did not differ between intact and burnt areas (area, *F*_1,20_ = 1.69, *P* = 0.21; interaction, *F*_1,20_ = 0.45, *P* = 0.51).

**Figure 6:**
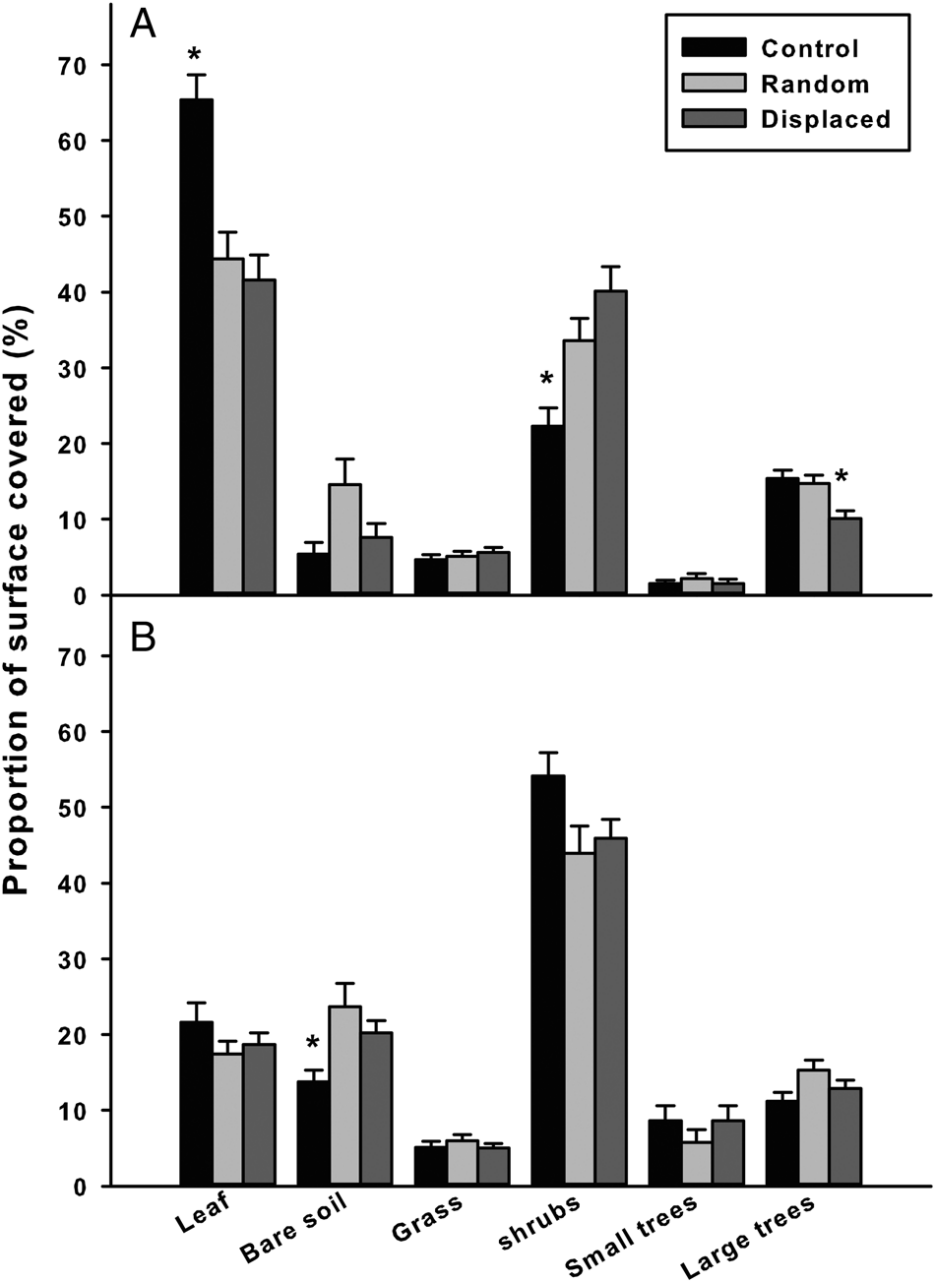
Mean (±1 SE, *n* = 6 per group) microhabitat use (expressed as a percentage) by control (black bars) and displaced (dark grey bars) radio-tracked tortoises in intact (**A**; top graph) and burnt areas (**B**; bottom graph). Significant selection was evaluated as high (preference) or low (aversion) compared with randomly sampled microhabitats (Random; light grey bars). *Statistically significant differences (Wilcoxon test *P* < 0.05; see main text).

We found several differences between randomly sampled habitats and the microhabitats used by the radio-tracked tortoises, suggesting that tortoises selected specific microhabitats (Fig. 6). In the intact area, control tortoises often selected shaded microhabitats with leaf litter (65.4%; *W* = 3373.5; *P* < 0.01) and they avoided shrubs (22.3%; *W* = 1728.0; *P* < 0.01). Displaced tortoises tended to avoid large trees, mostly in the intact area (10.1%; *W* = 1822.0; *P* < 0.01). In the burnt area, control tortoises were often observed in shrubs (54.1%; *W* = 1505.5, *P* < 0.05) and they avoided bare ground (13.8%; *W* = 946.5; *P* < 0.05).

Mean body temperatures did not differ between control and displaced individuals or between individuals in burnt and intact areas (displacement, *F*_1,17_ = 1.15, *P* = 0.30; area, *F*_1,17_ = 2.25, *P* = 0.15; interaction, *F*_1,17_ = 0.44, *P* = 0.52; Table 2).

**Table 2:** Summarized mean temperatures (±1 SD) of the four experimental groups of tortoises radio-tracked in spring 2009, between 10 April and 20 May (three loggers did not function well, reducing the initial sample size in two groups)

Area	Status	Shell temperature	*n*
Intact	Control	18.2 ± 0.7	6
Intact	Displaced	17.5 ± 0.9	6
Burnt	Control	19.1 ± 0.7	4
Burnt	Displaced	19.8 ± 1.1	5

## Discussion

The 2003 fire, classified as one of the strongest ever recorded in southeastern France, carbonized almost all of the vegetation in its path, and its effects on vegetation were still marked 6 years later (Fig. 1). This is consistent with fire-recovery timing in Mediterranean regions (Prométhée, 2010). The tortoises that survived the highly destructive fire could have experienced the following consequences: (i) permanent physiological and morphological trauma; (ii) a burnt habitat, with very sparse herbaceous or shrub vegetation, causing possible food and shelter shortages; and (iii) a drastically modified landscape during several years, posing possible complications to the individuals (e.g. different vegetation, odours) during their day-to-day life. Therefore, we expected strong negative impacts on tortoises.

### Tortoises in intact vs. burnt habitats

The main outcome of this study is that fire-surviving tortoises that remained in habitats ravaged by fire had body condition similar to those tortoises in unburnt habitat. This counter-intuitive result was supported by several lines of evidence and results, which showed that migration and dispersal processes were not mixing tortoises from different areas, at least 6 years after the fire. First, the homing experiment revealed that the tortoises sampled in 2009 in each area were resident and not vagrant individuals, in accordance with the home fidelity exhibited by tortoises (Chelazzi and Francisci, 1979), and thus were representative of the intact and burnt habitat, respectively. Radio-tracked control tortoises remained in their subarea, whereas most displaced tortoises travelled large distances in straight lines to return home. Second, individually marked tortoises were systematically recaptured in the same area over time (B. Livoreil, unpublished observations). Third, we did not find individuals (e.g. road-kill, radio-tagged) crossing the road that separates the intact and burnt areas. Other results from the homing experiment suggest that tortoises coped equally well in intact and fire-impacted habitats.

### Determinants of fluctuations in body condition

The results for body condition and temperature indicate that the tortoises from the burnt area did not face specific difficulties in finding food and thermal resources compared with individuals living in the intact area (Fig. 3). Indeed, marked inter-annual variations in mean body condition were observed, but with no significant difference between the two areas. It is likely that in both areas, annual climatic conditions (e.g. ambient temperatures, rainfall) influenced food and water availability, energy budgets and, thus, individual body condition (Nagy and Medica, 1986). Notably, 2003 and 2005 were two very hot years in France, characterized by marked spring and summer droughts (Soubeyroux *et al.*, 2011), and the mean body condition of the tortoises was particularly low during these two years (Fig. 3). Results from the homing experiment provided further support that habitat differences between intact and burnt areas were not the main drivers of the fluctuations in body condition. Indeed, we did not find any significant differences in mean body condition or in mean body temperatures between the four experimental groups of tortoises. This suggests that the wide range of microhabitats (e.g. herbaceous layer, shrubs and trees) available in each habitat, although different in the intact and burnt area, enabled tortoises to find adequate shelter and, probably, to thermoregulate reasonably well (i.e. there were no indications of heat-stressed or frozen tortoises). Taken together, these results suggest that tortoises adjusted well to the burnt habitat. Following fire, in late August, the rapid growth of herbaceous vegetation after rain in late summer may well have provided sufficient food to surviving tortoises.

### Comparison with other studies

A lack of impact of fire on body condition and fecundity has also been reported for Agassiz's desert tortoise (*Gopherus agassizii*), a species that can retreat into deep burrows to avoid fire (Lovich *et al.*, 2011). In addition, prescribed fires where the main combustible vegetation is the herbaceous cover are not necessarily destructive, especially for burrowing chelonians (Greenberg *et al.*, 1994; Hermann *et al.*, 2002; Yager *et al.*, 2007; Lovich *et al.*, 2011). In contrast, strong Mediterranean forest fires are often highly destructive (Syphard *et al.*, 2009), notably for tortoises belonging to the *Testudo* genus, because they do not burrow (Hailey, 2000; Cheylan, 2001; Popgeorgiev, 2008; Couturier *et al.*, 2011; Sanz-Aguilar *et al.*, 2011). Slow-moving and non-burrowing animals cannot easily escape heat waves and heavy smoke. In the present study, many individuals were found burnt or asphyxiated in the burnt area; 30 recently dead burnt tortoises with a complete shell and seven dead individuals without burn scars (B. Livoreil, unpublished observations). Overall, although fires can have a strong immediate negative impact on tortoise populations in Mediterranean regions, this does not mean that surviving individuals face specific (or unsurmountable) difficulties after fire.

### Limitations and conclusion

Our study focused on adults. However, neonates and juveniles are extremely elusive (Ballouard *et al.*, 2013; Couturier *et al.*, 2013) and they are more vulnerable to environmental perturbations than adults (Hailey, 2000). Due to their small body size, young tortoises are particularly sensitive to overheating, dehydration and predation (Barje *et al.*, 2005). Maturity requires ∼8–12  years in *Testudo* species (Cheylan, 1981, 2001; Lagarde *et al.*, 2001; Díaz-Paniagua *et al.*, 2001). Overall, possible short- (direct fire mortality and predation) and long-term impacts (low recruitment) of fire on populations through the effect on juveniles was not examined, and this issue should be evaluated in the future.

Despite this important limitation, our results have important management implications. For example, tortoises walking in burnt landscapes provide the deceptive impression that they may not survive without help, possibly triggering inappropriate action, such as translocations to unburnt areas, at least by the general public (B. Livoreil, unpublished observations). We suggest promoting (e.g. via technical notes, media campaigns) a different option, i.e. tortoises surviving fire should be left in their home range, even if the habitat is severely burnt. Indeed, inappropriate translocations may elicit homing behaviours, increase mortality risks due to hazardous displacements (e.g. crossing road or obstacles; Pérez *et al.*, 2004; Golubović *et al.*, 2013) and hamper population recovery. Population augmentation programmes (e.g. using captive breeding) should be tested to compensate for mortality caused by fire. Although this option has not been evaluated, successful translocations of adults have been performed in tortoises (Field *et al.*, 2007; Drake *et al.*, 2012). Given that fire destroys thick bushes and, thus, may expose juveniles to specific mortality, a possible efficient practical action would be to increase refuge availability in order to promote juvenile survival. Refuge availability can be reduced by strong and/or subsequent fires or by the rarefaction of shrubs caused by closing of the habitat. Promoting the growth of thick bushes (e.g. forest clearing, plantation) or setting up artificial refuges suitable for small tortoises should be tested (Ballouard *et al.*, 2013).
